# Acute Care and Medication Management in Older Adults

**DOI:** 10.3390/jcm10020166

**Published:** 2021-01-06

**Authors:** Patrick Manckoundia, Alain Putot

**Affiliations:** 1 Department of Geriatrics and Internal Medicine, Hospital of Champmaillot, Dijon Bourgogne University Hospital, 21079 Dijon, France; alain.putot@chu-dijon.fr; 2 INSERM U-1093, Cognition, Action and Sensorimotor Plasticity, University of Burgundy Franche-Comté, 21000 Dijon, France; 3 Physiopathologie et Epidémiologie Cérébro-Cardiovasculaires (PEC2), EA 7460, University of Burgundy and Franche Comté, 21000 Dijon, France

The number of healthy and frail older adults is increasing worldwide, especially in industrialized countries, as a result of the increase in life expectancy. Indeed, medical advances and improvements in hygiene and living conditions have led acute diseases that were once fatal to be less so, leading to an increase in the frequency of chronic diseases.

Healthy life expectancy is one of the major public health issues of the moment. Increasing healthy life expectancy requires a good understanding of the specific conditions found in older adults, whether they are frail or not. It is essential to fully understand, among other things, the acute diseases of the elderly and very elderly to provide them with the most suitable treatments. Ensuring appropriate management will help to avoid a number of pitfalls including undertreatment and overtreatment. Despite the specific aspects of older patients in terms of both semiology and therapeutics, still relatively few publications address acute care in this population.

This Special Issue of the *Journal of Clinical Medicine (JCM)* offers teams with recognized expertise in the care of the elderly the opportunity to publish quality research work on acute care and/or drug management in this population. This Special Issue is open to varied topics in these two fields (acute care in older adults and medication management in older adults). Original articles on age-related atypical presentation, diagnostic tools and the acute management of common conditions in older patients (including acute infections and acute cardiovascular disorders) are particularly welcome.

In closing, in this issue, we challenge you to provide your best visionary science to help advance the management of acute diseases in the elderly population.

